# Media Bias in German News Articles: A Combined Approach

**DOI:** 10.1007/978-3-030-65965-3_41

**Published:** 2020-12-09

**Authors:** Timo Spinde, Felix Hamborg, Bela Gipp

**Affiliations:** 5 University of Sydney, Sydney, NSW Australia; 6 Monash University, Clayton, VIC Australia; 7 University of Bari Aldo Moro, Bari, Italy; 8 University of Bari Aldo Moro, Bari, Italy; 9 University of Guelph, Guelph, ON Canada; 10 University of Caen Normandy, Caen, France; 11 University of Pisa, Pisa, Italy; 12 Norwegian University of Science and Technology, Trondheim, Norway; 13 University of Porto, Porto, Portugal; 14 UPC BarcelonaTech, Barcelona, Spain; 15 University of Porto, Porto, Portugal; 16 Fraunhofer IAIS, St. Augustin, Germany; 17 Royal Holloway University of London, Egham, UK; 18 University of Lisbon, Lisbon, Portugal; 19 University of Bari Aldo Moro, Bari, Italy; 20 University of Lisbon, Lisbon, Portugal; 21 University of Bari Aldo Moro, Bari, Italy; 22 ICAR-CNR, Rende, Italy; 23 University of Naples Federico II, Naples, Italy; 24 University of North Carolina, Charlotte, NC USA; 25 Australian National University, Canberra, ACT Australia; 26 Leibniz University Hannover, Hannover, Germany; 27 Technical University of Dortmund, Dortmund, Germany; 28 University of Southern Denmark, Odense, Denmark; 29 University of Pisa, Pisa, Italy; 30 Warsaw University of Technology, Warsaw, Poland; 31 ISTI-CNR, PISA, Italy; 32 Berlin Institute of Technology, Berlin, Germany; 33 Berlin Institute of Technology, Berlin, Germany; 34 Norwegian University of Science and Technology, Trondheim, Norway; 35 University of Konstanz, Konstanz, Germany; 36 University of Wuppertal, Wuppertal, Germany

**Keywords:** Media bias, News slant, News bias, Content analysis, Frame analysis

## Abstract

Slanted news coverage, also called media bias, can heavily influence how news consumers interpret and react to the news. Models to identify and describe biases have been proposed across various scientific fields, focusing mostly on English media. In this paper, we propose a method for analyzing media bias in German media. We test different natural language processing techniques and combinations thereof. Specifically, we combine an IDF-based component, a specially created bias lexicon, and a linguistic lexicon. We also flexibly extend our lexica by the usage of word embeddings. We evaluate the system and methods in a survey (N = 46), comparing the bias words our system detected to human annotations. So far, the best component combination results in an F$_{1}$ score of 0.31 of words that were identified as biased by our system and our study participants. The low performance shows that the analysis of media bias is still a difficult task, but using fewer resources, we achieved the same performance on the same task than recent research on English. We summarize the next steps in improving the resources and the overall results.

## Introduction

Media bias, i.e., slanted news coverage, can change the public opinion on any topic heavily [5]. Many approaches to identify such bias exist, however, no automated methods aiming to identify bias in German news texts are available. The objective of this work is to propose, implement and evaluate a system capable of detecting bias words in German news articles. The key contribution of this poster is our media bias identification approach, which includes five components: (i) An IDF-based component, which utilizes word frequencies over a set of documents. (ii) A sentiment-based component using multiple dictionaries. (iii) A component that uses a dictionary of bias words based on semantic models. Two other components that have not yet been implemented are (iv) a component that uses SVM with cues of historical linguistic development, and (v) a network analysis component. Moreover, we provide a summary of characteristics of sentiment in German language and the cultural development of words in specific classes, such as pejorative derivatives, from a linguistic perspective.

The research described in this paper is based on a recent poster publication [17]. In contrast to the poster, the paper at hand elaborates in more detail on the methodology, results, especially considering the single components, and future improvements of our system.

The main shortcomings of prior work are a dependency on manually created resources, a small number of polarity categories and a focus on only specific topics. First, some of these methods identify media bias using predefined dictionaries, requiring manual and effortful creation and adaption. Second, the possible emotional influence of the detected bias words has not been analyzed on a computational scale. Third, limited research has been conducted on the combination of existing approaches. Except from component (v), all methods mentioned above have already been implemented in other research, but we are especially taking an attempt in combining them.

In Sect. 3, we describe the five components in further detail. Finally, we show the evaluation methodology and offer an outlook on future work.

## Related Work

We first describe key concepts from linguistics, relevant in the context of media bias and framing. Then we give a brief overview of related methods that aim to identify media bias in news items.

### Linguistics

Linguistic cues are important properties when identifying either framing (a particular point of view linked to subjective words or phrases) or epistemological bias (subtly focusing on the believability of a proposition) [16]. While a summary of cues is shown in [11], not all such resources are available in German.

Word embeddings can be used to find semantically similar words for any given word [6]. For example, in a word embeddings space, the vectors of the following words would typically be close to each other: *Flüchtling* (refugee), *Migrant* (migrant), *Asylant* (Asylum seeker) or *Geflüchteter* (displaced person). The words are, even though legally not completely synonymous, very similar.

### Media Bias Detection Systems

In the following, we give a brief overview of relevant media bias detection systems. Linguistic cues are not the only way to solve the task: multiple approaches exist, mainly devised in computer science. A first way of identifying media bias is proposed by Ogawa et al., who use a stakeholder mining mechanism trying to analyze bias backgrounds [15]. The result of the analysis is a relationship graph which groups stakeholders who share mutual interests (and generally describes their interests), which can be especially interesting for another network analysis.

In 2013, Recasens et al. proposed an approach to identify bias words in Wikipedia [16]. They compiled a list of helpful linguistic resources and utilized them as features to detect the bias words from Wikipedias’ revised sentence history. Baumer et al. developed a prototype to identify framing [2]. The linguistic components they classified as “dictionaries” are what Recasens et al. proposed, but extended by features of theoretical literature on framing. Hamborg et al. proposed an approach that aims to identify bias by word choice and labeling (see the example in Sect. 2.1) [6]. They used word embeddings to resolve broad coreferences across a set of news articles reporting on the same event.

Hube et al. addressed biased language statements in Wikipedia articles [8]. Their approach is mainly based on building a context-related set of word semantics, which identifies the relevant language in a certain topic sphere. For that, they utilized a right-leaning adaptation of Wikipedia, Conservapedia, to train word embeddings. With these, they manually selected 100 potential bias words and computed for every word the 20 most similar words, to create a certain bias word dictionary. With this resource, they then identified biased sentences by calculating bias word ratios and contexts. By furthermore adding the linguistic features by Recasens et al. [16], they achieved an F$_{1}$ score of 69%. Their findings also suggested that, on crowdsourced evaluation data, the bias words were only very helpful in finding the most biased sentences, which might be due to the Conservapedia database being rather extreme.

The main shortcomings of prior work are a dependency on manually created resources, a small number of polarity categories, a focus on only specific topics, or a non-scalable evaluation. First, some of these methods identify media bias using predefined dictionaries or grammatical features, requiring the manual and effortful creation and adaption of dictionaries. Second, the possible emotional influence of the detected bias words has not been analyzed on a computational scale. Common polarity features only consist of three categories positive, negative, and neutral. Third, limited research has been conducted on methods that combine existing approaches, leaving out one possibly huge way to further overall performance improvement. Lastly, a primary and well-surveyed common data set to address all of these word- and sentence-oriented bias types could make a more reliable evaluation possible. By addressing the previously mentioned issues, the analysis of media bias using other methods could benefit: e.g., network analysis provides promising practices to model and visualize the relations and underlying information of text documents, enabling statistical modeling of media bias [13]. Section 3 describes our approach, which addresses these four issues. It is not dependent on single topics or resources and will be scalable to any sort of related task.

## Methodology

**Fig. 1.**
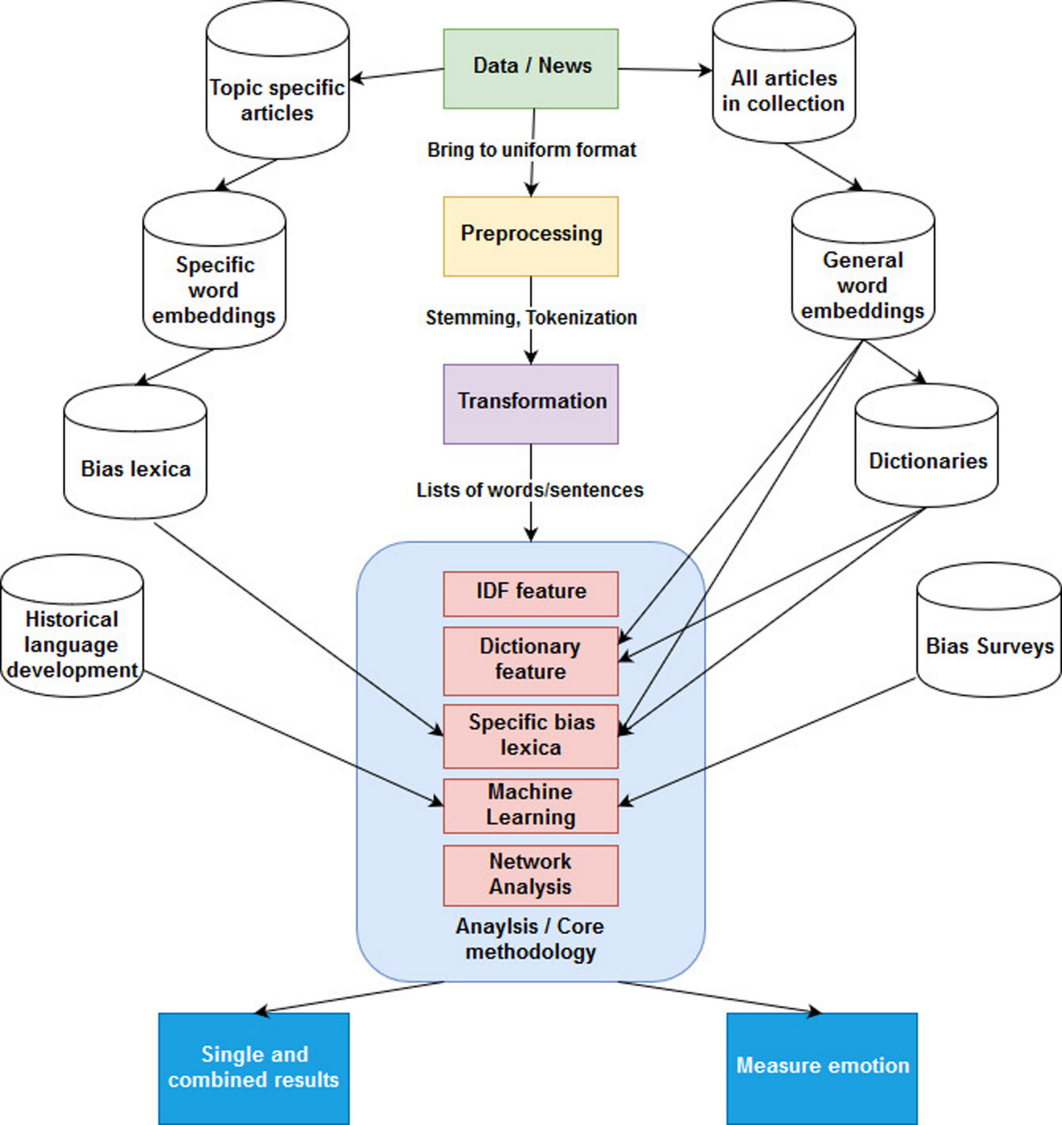
System architecture (Color figure online)

The methodology proposed in this paper consists of different steps, which are depicted as colored boxes in Fig. 1.

We have collected news articles from four German news outlets, *Süddeutsche Zeitung* (65,000 articles), *TAZ* (500,000), *Südkurier* (286,700), and *BILD* (2,000 articles reporting on the refugee crisis). To enlarge the data set for the training of word embeddings, we used a collection of articles by Bojar et al. [3], which contains almost 90M sentences from over 40 sources, including the *Augsburger Allgemeine* and *Der Westen*. We preprocessed all files into a uniform format. To train our model of word embeddings, we used all articles and the collection of sentences and compared which words are more likely to appear.

The automated analysis workflow consists of five components, of which the following three are implemented in our prototype: an IDF-based component (based on the idea by Lim et al. [11]), a combined dictionary-based component (based on the idea by Recasens et al. [16]), and a component based on semantically created bias dictionary (based on the idea by Hube et al. [8]). We experimented with different combinations of these components, e.g., to determine if a word was identified as biased by one component but not by the other (if so, we classified it as bias word). One of the two components that are not yet implemented will use SVM to analyze historic linguistic cues. Apart from connotation, context and emotion, some words have developed linguistic patterns playing a role in gender discussions, but also in general sentiment. The German word *Flüchtlinge* is one example. Its general impression is influenced by the derivational component *-ling*, which frequently is and has been origin for ameliorating replacement constructions, e.g. *Sonderling*, *Schönling* or *Schwächling*. Official discussions, however, lead into the direction that such derivatives should generally be replaced by participle derivative constructions like *Geflüchtete*[7]. Our literature review yielded that no central collection of such rules or any large scale analysis of their real effects exists. Evaluating their impact and gathering similar rules will be a major future task. The second component that is not yet implemented relies on network analysis. Network structures can effectively represent not only the documents or news themselves but also model relations and correlations. A variety of nodes, edges, and attributes come to mind, such as newspapers, authors, emotional scores, bias words, content, year or time of publication and topic. With a sufficiently large data set and further reliable methodology to detect the actual values, topic- and context-dependent patterns could be modeled. Inherent characteristics would be centrality, clustering, and betweenness.

The first component uses IDF scores to measure whether a term is common or rare across the corpus. Thus, it serves as a dictionary-independent component to identify bias words. This way, we aim to find rare words in the collection of articles reporting on the same event. Lim et al. propose that, for such a set of news, words with high IDF scores are most likely to be biased words [11]. IDF scores were first calculated among the whole set of articles to be analyzed. We clustered the documents into the even more similar ones by using affinity propagation (which is a state-of-the-art clustering algorithm [20]), and analyzed again. This approach has not been applied in the media bias context within other literature. Therefore, we evaluated the first experimental results for both combinations of steps: IDF scores over all articles and of only the most similar ones due to different thresholds.

We based the second component mainly on a linguistic lexicon, containing factive and assertive verbs, entailments, hedges, subjective intensifiers, and one-sided terms [16]. We use the German Linguistic Inquiry and Word Count (LIWC) dictionary, published by Wolf et al. [22]. As especially slang and sociolect words are excluded, we include a separate dictionary by El-Assady et al. [9]. In a final step, we also extend the dictionary by assertive verbs, scraped from two sources: The *Online-Wortschatz-Informationssystem Deutsch* (OWID), a dictionary for corpus-based lexicography of contemporary German [14], and a collection by Edeltraud Winkler from 2007 [21].

With these resources, words were classified as bias words if they matched with any dictionary entry. To improve performance, words were also seen as biased if one of their two most similar terms, as modeled by the word embeddings described before, matched. The dictionary, primarily because it is based on the LIWC, gives an excellent opportunity to measure emotional, social, or psychological reactions to words [10].

**Fig. 2.**
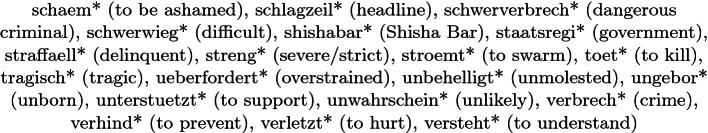
Random sample of the newly built bias lexicon

The third component uses a topic-specific bias dictionary, based on a separate data set and word embeddings. To create such a dictionary, seed words are extracted and used to retrieve other bias words. The idea is, as shown in [8], to use word vectors from documents which “are expected to have a high density of bias words.” For each 10-word batch in an initial manually selected list, the 20 most similar words are retrieved and again merged into one list, which hence contains 200 higher potential bias words. This process is then iterated a second time: the 200 words are used as new seed words to extract another 20 most similar words among batches of 10, which leads to an overall of 400 bias words. The full bias dictionary is then added to the dictionary described in the previous section. The overlap was 42%, so most of these bias terms were not previously included. The word embeddings for this first prototype were based on a 2000 article collection from the *Bild* news outlet, which uses rather strong language and is hence more likely to contain bias words than a more neutral medium [1]. A random 20 word sample of the lexicon can be seen in Fig. 2. The German words are all given in their stemmed version, with an English translation to give a better impression of the meaning. Even though there exist some exceptions, most of the words seem very plausible for inducing bias.

## Evaluation

To evaluate the approach, it was necessary to build a ground-truth data set which exhibits words that humans identify as bias words in a text with news characteristics. We conducted a test, in which we asked 46 participants (mostly students aged between 15–30 years, of balanced gender, from various study programs but without linguistic background, and consuming news daily while not intentionally comparing different media sources) to read two or three news articles, depending on the text length. The same group of articles has been shown to four persons. For each text, we asked them to highlight bias words, i.e., words they “felt were inducing an assessment.” We used a data set of 40 manually selected articles from *Bild*, *Junge Welt*, *Frankfurter Allgemeine Zeitung*, *Frankfurter Rundschau*, *Compact Online*, *PI-News*, *NachDenkSeiten* and *RT online*, all published from 2015 to the end of 2018. The participants made 718 annotations in total. Only words that were at least mentioned by 2 of the 4 persons in each group were kept, which reduced them to 432 bias words used in the evaluation. The data set was then used to determine the accuracy of the different components combined and individually. As baseline components, we used an IDF component and random guessing that selected every word as bias word with a 50% chance. The extended dictionary component, supplemented by the newly created bias dictionary, performed best (F$_{1}$ = 0.31). It outperformed the pure IDF component by 0.14 and random guessing by 0.26. In similar work by Recasens et al. [16], they achieved an overall F$_{1}$ score of 0.34, however using the more sophisticated dictionaries that are available for English language. On nouns, verbs, and adjectives, it correctly identified around half of the words, even though false positive rates for nouns and verbs were still relatively high. For adjectives, we achieved an F$_{1}$ score of over 0.40. General word embeddings mostly did not result in any improvement for all of the components and their combinations. It seems that the assumption that words similar to bias words are naturally also bias words did not hold. Overall F$_{1}$ score results are shown in Table 1. It will be a major future research direction to not only improve the components but also to create a larger and extensively tested evaluation data set.

**Table 1.**
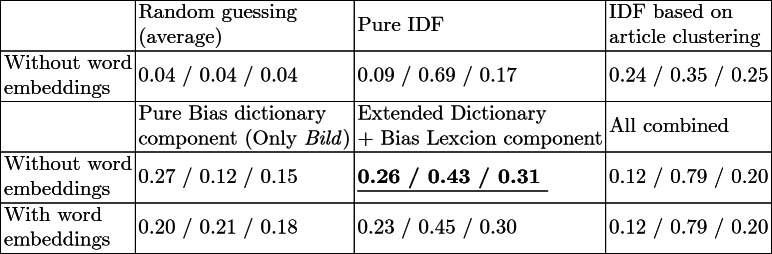
All evaluation results of the bias word detection, with precision/recall/F$_{1}$ score per cell

We applied the best performing component in three small case studies to give insights into the potential of the overall approach. While analyzing newspapers within three topics (refugees in general, the 2018 Chemnitz protests [4] and the refugee politics of Viktor Orbán [19]), our proposed approach was able to identify general tendencies in political classifications of German news media [12].

From a qualitative perspective, some of the words in our lexicon were never identified as biased. The verb ‘to understand’, given in the lexicon sample in Fig. 2, is a good example. It appears that, even though we introduced many words that can be seen as potential bias words, this does not apply for all of them. As in the work by Hube et al. [8], we did not filter the words we added using the methodology in our third component. In the future, we plan to analyze the characteristics of each of the newly found potential bias word, to reduce the number of false positives.

## Conclusion and Outlook

This paper proposed a work-in-progress approach to identify media bias words in German news texts. Moreover, the approach capably identifies emotion markers. The approach currently implements three components: an IDF-based component, selecting terms based on their frequency among a given corpus; a dictionary-based component, for which we merged and linked four sources of emotional and linguistic terms; and lastly a topic-dependent bias word dictionary that we created using word embeddings, calculated over a set of articles from the newspaper *Bild*. We plan to make our code and resources publicly available under an open-access license, but are currently verifying licenses of the included dictionaries.

We have compared the performance of our components with each other, based on an evaluation data set created using a bias word survey with 46 participants, each of them reading and highlighting words in up to three articles. We find that the dictionary component, combined with the topic-dependent bias word dictionary, performed best (F$_{1}$ = 0.31, P = 0.43, R = 0.26). When considering only adjectives, F$_{1}$ was 0.41. Integration of word embeddings did not lead to higher accuracy, i.e., F$_{1}$ = 0.30. Furthermore, we conducted a case study, which showed that the emotional detection function of the approach could already detect presumed differences between major German newspapers, such as *Bild*, *Frankfurter Rundschau* or *TAZ*. Despite the difficulty of detecting media bias, even for humans, we think our approach is a first step towards automatically analyzing bias in German media. Upcoming research will focus on improving the underlying model by enlarging the dictionary, adding more bias dictionaries for individual newspapers, training more reliable word embedding models, gathering a larger amount of data and especially integrating context. A more extensive evaluation, gathering a more precise ground truth, will also be essential. We will test two features for further improvements: machine learning using human bias classifications and historic rules of language development as well as a network analysis incorporating the context of documents. It could also be interesting to try to find out how we could show and visualize our results best [18]. Ultimately, our goal is to identify bias wording automatically and to understand the underlying emotions in a greater context.
